# Comparing fresh root yield and quality of certified and farmer-saved cassava seed

**DOI:** 10.1016/j.cropro.2024.106932

**Published:** 2025-01

**Authors:** Juma W. Yabeja, Mkabwa L.K. Manoko, James P. Legg

**Affiliations:** a International Institute of Tropical Agriculture (IITA), Dar es Salaam, Tanzania, Box 34441, Dar es Salaam, Tanzania; b College of Agriculture and Food Technology (CoAF), University of Dar es Salaam, Tanzania, Box 23456, Dar es Salaam, Tanzania

**Keywords:** Certified seed, Economic benefit, Farmer-saved seed, CBSD, Yield, Tanzania

## Abstract

Formal systems supporting the delivery of high-quality cassava seed are being established in several key cassava producing countries in Africa. Questions remain, however, about the value of certified cassava seed when compared to seed which is recycled multiple times, which is standard farmer practice. A study was therefore conducted to compare fresh cassava root yields of high-quality seed (HQS) versus farmer-saved (recycled) seed (FSS) for three widely grown improved cassava varieties in Tanzania namely: *Mkuranga1*, *Kiroba* and *Mkombozi*. Field experiments were established in two sites in different agricultural zones: Mkuranga (Coast Zone) and Maruku (Lake Victoria Zone). Four HQS sources (pre-basic, basic, certified, quality-declared), collectively referred to as HQS, were compared with FSS with respect to cassava brown streak disease (CBSD) foliar and root incidences, fresh root yield, marketable fresh root yield, and usable fresh root yield for each variety in the two locations. Results showed that foliar CBSD incidence in FSS was significantly greater than it was for HQS in *Mkuranga1* and *Kiroba* varieties but not for *Mkombozi*. CBSD root incidence was on average six times more in FSS than in HQS. When comparing FSS with the specific certified seed treatment (CS), 25.8% of the roots were unusable due to CBSD root necrosis for FSS, compared to only 3.7% for CS. CS gave an overall fresh root yield which was 7.5 t/ha more than FSS, representing an 80.6% increase. Yield benefits derived from planting HQS were similar for *Kiroba* (+80.7%), *Mkombozi* (+81.3%) and *Mkuranga1* (+79.5%), as well as across each of the four HQS classes. When also considering losses arising from severe CBSD root necrosis, the overall yield benefit arising from using CS when compared to FSS was 135%. The average estimated income gain for this increase was US$ 2279/ha, which is many times the estimated cost of obtaining certified seed. These results highlight the value of high quality seed systems and the potential gains that farmers can realize from planting high quality certified seed rather than recycling existing crops.

## Highlights

• High quality cassava seed (HQS) performed better than farmer-saved seed (FSS).
• Cassava brown streak disease root incidence was 6 times greater in FSS than in HQS.
• Fresh root yield of HQS was 80.7% greater than for FSS of the same variety.
• 25.8% of FSS roots were unusable due to cassava brown streak root necrosis.
• The overall yield benefit of certified seed compared to FSS was 135%.

## 1 Introduction

Cassava (*Manihot esculenta* Crantz) is a vegetatively propagated crop, used as a staple food and income source for almost 800 million people globally (Burns et al., 2010; Patil and Fauquet, 2015). Currently, the global total annual production is more than 330 million tonnes, roughly 63% of which is in Africa (FAOSTAT, 2022). Tanzania is one of the most important cassava producing countries in Africa. However, although the yearly average production is almost 6.4 million tonnes, cultivated over an area of 993,500ha, the productivity is low, with an average yield of 6.4 t/ha (FAOSTAT, 2022), which is much less than the potential yield of 25-30 t/ha (Lebot, 2009). This low productivity remains a major challenge which hinders food security and the prosperity of smallholder farmers. One important reason for low cassava productivity is the low adoption level of certified seed (=planting material) (Van den Broek et al., 2015). Some of the reasons for this include: limited access to certified seed; unaffordable seed prices; volatile market prices for cassava produce as well as limited awareness about the value of certified seed (Bold et al., 2017; Wossen et al., 2020). Consequently, about 80–90% of smallholder farmers in South Asia and Sub-Saharan Africa opt to recycle their seeds (Mula et al., 2013). However, continual recycling of seed, particularly that of vegetatively-propagated crops, often results in reduction of the quality of the seed due to the build-up of disease-causing pathogens (Shirima et al., 2019). For cassava, persistent recycling of seed results in the propagation of two important viral diseases – cassava brown streak disease (CBSD) caused by cassava brown streak ipomoviruses (CBSIs) (Mbanzibwa et al., 2009; Winter et al., 2010) and cassava mosaic disease (CMD) caused by cassava mosaic geminiviruses (Bock and Woods, 1983). The viruses causing these diseases are transmitted by the insect vector, *Bemisia tabaci* (Dubern, 1994; Maruthi et al., 2005, 2017) and they are also spread through the use of infected cuttings as planting material (Legg et al., 2011; Nichols, 1950). According to Shirima et al. (2019), sustained recycling leads to the degeneration of cassava seed which adversely affect root quantity and quality due to root necrosis, making the roots inedible (Gondwe et al., 2003). Yield losses caused by these viral diseases typically range from 30 to 100% (Kawuki et al., 2016; Okonya et al., 2019), and Africa-wide losses worth more than US$1 billion yearly (Legg et al., 2006). Losses due to CBSD in Tanzania alone have been estimated at US$ 51 million annually (Ndyetabula et al., 2016).

The use of certified seed of any food crop, irrespective of other agronomic factors, is widely recognized as an important factor that leads to increased yield and higher market values for crop products (Dogbe et al., 2014; Guei et al., 2011; Louwaars and De Boef, 2012; Poonia, 2013). Estimates of yield increments arising solely from the use of certified seeds are: 5–20% (Sahu et al., 2020), 15–20% (Poonia, 2013), 20–25% (Hasanuzzaman, 2015) and 47% (Asiedu et al., 2007). However, the yield quantification data mentioned are exclusively limited to cereals and legumes and there is no comparable information available for cassava. Efforts have been made over several decades to develop a modernized seed system for cassava in Tanzania (Douthwaite, 2020), which currently comprises four classes of seed (= planting material), including pre-basic, basic, certified and quality declared seed (QDS) (Legg et al., 2022), collectively referred to here as ‘high-quality seed’ (HQS). Although it has been assumed that HQS, managed to minimize levels of disease infection, will provide greater yields than farmer saved seed (FSS) of the same variety, there is presently no published evidence to confirm this. The current study was undertaken to address this knowledge gap. Key objectives of this research were to determine the difference in disease infection in farmer-saved seed compared with HQS, and most importantly, to quantify the yield change resulting from planting certified seed. Although Tanzania's environmental context has some unique characteristics, these results are expected to be indicative for all cassava-growing environments where CBSD is a major constraint, notably including large parts of east, central and southern Africa.

## 2 Materials and methods

### 2.1 Study area

On-station field experiments were conducted at two sites: Mkuranga Experimental Research Station (Altitude 126 m above sea level, latitude −7.1402, longitude 39.1964) in Mkuranga District, Coastal Region, and Maruku Research Centre (Altitude 1531 m above sea level, latitude −1.4206, longitude 31.7761) in Bukoba District, Kagera Region, in Tanzania. Maruku experiences a bi-modal rainfall pattern, with average annual rainfall of approximately 2,000 mm and a temperature range from 17 to 26 °C. Mkuranga has a similar bimodal rainfall pattern to that of Maruku, with average annual rainfall of approximately 800 mm and a temperature range from 19 to 32 °C.

### 2.2 Experimental design

The study was conducted over two years across the two sites. At the Mkuranga site, the first planting season was 2020/2021 and the second season was 2021/2022. At Maruku, the first planting season was 2021/2022 and the second season was 2022/2023. At both sites, a randomized complete block design (RCBD) was used to establish the field experiments. Cassava varieties and their corresponding seed classes were the two treatment factors used in comparing root yield and CBSD incidences for HQS and FSS. The choice of cassava varieties for the field experiments depended on their availability across all of the four seed classes (Pre-basic – PB, Basic – B, certified – C, and Quality Declared Seed – QDS) of the formal cassava seed system as well as in the FSS category from the informal cassava seed system. Based on these selection criteria, varieties *Mkuranga1* and *Kiroba* were obtained in Mkuranga and variety *Mkombozi* in Maruku. For Mkuranga, where two varieties were tested, the combination of two varieties and the five seed classes resulted in a total of ten treatment combinations (PB-Kiroba, PB-Mkuranga1, B-Kiroba, B-Mkuranga1, C-Kiroba, C-Mkuranga1, QDS-Kiroba, QDS-Mkuranga1, FSS-Kiroba and FSS-Mkuranga1). For Maruku, with one variety, there were five treatment combinations (PB-Mkombozi, B-Mkombozi, C-Mkombozi, QDS-Mkombozi and F-S Mkombozi). The treatments were replicated three times for each site. The individual plots for each treatment consisted of 42 plants, planted in 6 rows with 7 plants per row and all spaced at 1m × 1m.

### 2.3 Pre-planting evaluation of cassava planting material used for the field experiments

Cassava seed used for the field experiments was sourced from both HQS sources as well as uncertified FSS. However, for each planting season the planting materials were sourced from different fields. HQS for the three varieties was collected from PB, B, C, and QDS seed fields. However, FSS of the same varieties was obtained from fields of farmers who had not obtained HQS for at least four years (Table 1). This meant that FSS had been recycled for at least four years. A pre-assessment was conducted for the selected PB, B, C and QDS fields to ensure that levels of disease fell within the tolerance levels allowable by the formal certification guidelines, for example for CBSD, allowable foliar incidence levels are: PB – 2%, B – 4%, C – 7% and QDS – 10% (Legg et al., 2022). Furthermore, pre-assessment was also carried out for FSS to determine the CBSD level at the onset of the experiment. The disease assessment procedure followed the method used by the seed regulatory authority – the Tanzania Official Seed Certification Institute (TOSCI). This comprised the sampling of 200 plants per field, being made up of five randomly selected quadrats of 40 plants each. Once the field had been shown to conform to the required quality standards above, mature stems were collected at random. For FSS, farmers were requested to select cassava stems from their fields based on their normal seed selection practices. In each season, the protocols applied were the same to ensure that each season's seed sources conformed to the same quality guidelines.

**Table 1:** CBSD incidence (%) in HQS and FSS sources, and number of recycling rounds for FSS.

Site	Season	Variety	Pre-Basic	Basic	Certified	QDS	FSS (%)	FSS number of recycling rounds
Mkuranga	1	Kiroba	0.5	3	6	8	72.5	4
	1	Mkuranga1	0	0	0	0	77.5	5
								
	2	Kiroba	0	3.5	5	10	85.5	5
	2	Mkuranga1	0	0	0	5	94.5	6
								
Maruku	1	Mkombozi	0	0.5	1	2	71.5	5
	2	Mkombozi	0	0	0	0	68.0	6
Average			0.08	1.2	2.0	4.2	78.3	

### 2.4 Agronomic practices

#### 2.4.1 Characteristics of planting material and planting style

For all treatments, cassava stems were cut into pieces of approximately 20 cm long and with at least five nodes as prescribed in the official cassava seed quality standards (Legg et al., 2022). During planting, the cuttings were planted at an angle of about 45°, with the lower half of the cutting buried beneath the soil surface. Low (<10%) levels of CMD were present in source fields for each of the treatments, but the only disease with high levels of incidence in some source fields (FSS) was CBSD. Throughout the course of the experimental trials, the only important pest/disease which spread through trial plots was CBSD, and there were no other potentially confounding pests/diseases noted from source planting material used for either of the experimental sites. CBSD spread was recorded from FSS plots to HQS plots, particularly at the Mkuranga site. This was anticipated, and its effects on the results have been addressed in the discussion. This spread had a minimal influence on the overall results, notably since within-season infection by cassava viruses has a much lower impact on plant growth than initial infection through the cutting.

#### 2.4.2 Gap filling and field management

At one month after planting (1MAP), the experiments were inspected to determine the establishment rate. A small number of gaps were filled with saved stock of the same treatment categories to ensure that all experiments attained the intended plant population. The field experiments were kept weed-free by manual wedding and no chemical or organic fertilizer was applied throughout the experiments.

### 2.5 Data collection

#### 2.5.1 Foliar CBSD symptoms and whitefly abundance assessment

In all sites, foliar CBSD symptoms were assessed for all 42 plants in each plot and scored using a severity scale of 1–5 where: 1 = no apparent symptoms; 2 = slight foliar feathery chlorosis on <25% of leaves and no stem lesions; 3 = pronounced foliar feathery chlorosis on 25–50% of leaves, mild stem lesions, and no dieback; 4 = severe foliar feathery chlorosis on >50% of leaves, severe stem lesions, and no dieback; and 5 = defoliation, severe stem lesions, and dieback (Gondwe et al., 2003; Hillocks et al., 1996). CBSD symptoms were assessed monthly throughout the 12-month duration of each trial. As described by Sseruwagi et al. (2004), foliar CBSD incidence was calculated as the percentage of symptomatic plants out of the 42 assessed in each plot. Furthermore, adult whiteflies were counted on the undersides of the top five leaves of the tallest shoot of each of the 42 plants sampled per plot across the experimental sites.

#### 2.5.2 Marketable and non-marketable root numbers and fresh weight yield

In all sites, at harvest time (12 MAP), the 20 plants in the net plot (which excluded the 22 edge plants) were uprooted, and their roots were detached from the plants. Subsequently, the total number of cassava roots per plant was counted and grouped into two categories: marketable large roots and unmarketable small roots based on the farmers’ practice for distinguishing between the two. Normally, farmers select medium to large-sized cassava roots for market sale, whilst smaller roots which are difficult to peel and sell are retained for home consumption (Nzola et al., 2022). The total number of marketable and unmarketable roots was recorded for each plot. The total weight of roots in each plot was then measured to give the total fresh weight yield.

#### 2.5.3 CBSD root necrosis incidence, severity, and usable/unusable fresh root yield

In all sites, each harvested root from the net plot was transversely cut into five pieces at evenly spaced intervals to check for CBSD necrosis (Ndyetabula et al., 2016). The level of CBSD severity was assessed using a severity scale ranging from 1 to 5, where 1 stands for asymptomatic and 5 for severe CBSD necrosis as described by Hillocks et al. (1996). The CBSD necrosis data were used to categorize the roots into usable and unusable based on the CBSD scores. All roots that had a maximum severity score of 2 were considered usable while those with severity scores from 3 to 5 (even for a single slice per root) were considered unusable (Ndyetabula et al., 2016). These results were used to calculate the proportion of usable and unusable roots in each plot and these proportions were multiplied by the total fresh root yield for the plot to give the usable and unusable root yield values for the plot.

### 2.6 Data analysis

Descriptive statistics were used to summarize the data on disease scores to obtain disease incidences, which were subsequently used to plot line graphs. The non-parametric Kruskal-Wallis test was used to analyse differences in disease incidence levels (percentages) across the seed classes. Dunn's test was applied for pairwise comparison of differences in disease incidences across the seed classes. Parametric ANOVA statistics were used to analyse the yield data to test for differences in mean yield across treatments. Additionally, the Least Significant Difference statistic (LSD) was used for mean separation analyses of yield data. Whitefly abundances were compared using parametric t tests. All the statistical tests were executed using ‘R’ version 4.4.1.

## 3 Results

### 3.1 Foliar CBSD indicence in the seed source field and recycling period

For HQS categories, CBSD incidences in seed source fields ranged from 0 to 10% whilst for FSS they ranged from 68 to 94.5%, with average of 5 years of recycling (Table 1). CBSD incidences in the HQS categories were within the tolerance levels prescribed by the official quality standards (Legg et al., 2022). Across varieties, CBSD incidences from HQS were higher for *Kiroba* than for *Mkuranga1* or *Mkombozi*.

### 3.2 Foliar CBSD incidence and whitefly abundance

**Fig. 1:**
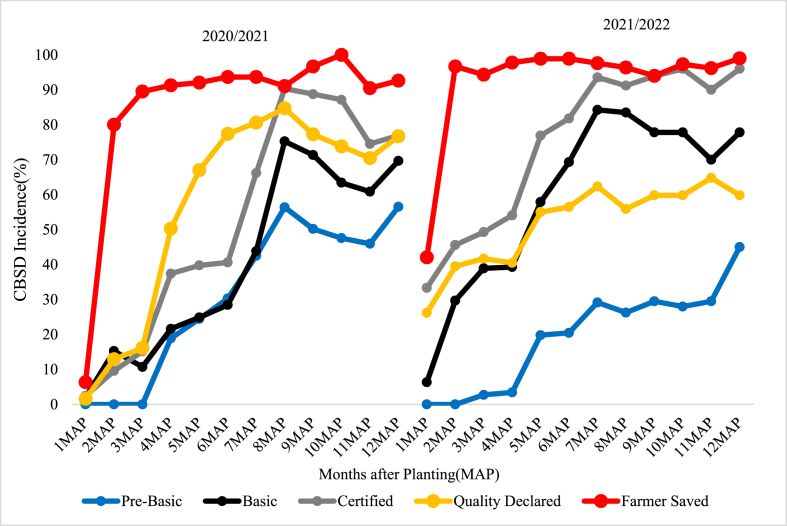
Foliar CBSD incidence recorded at monthly intervals in plots of Kiroba variety for two seasons at Mkuranga site, central-eastern Tanzania.

**Fig. 2:**
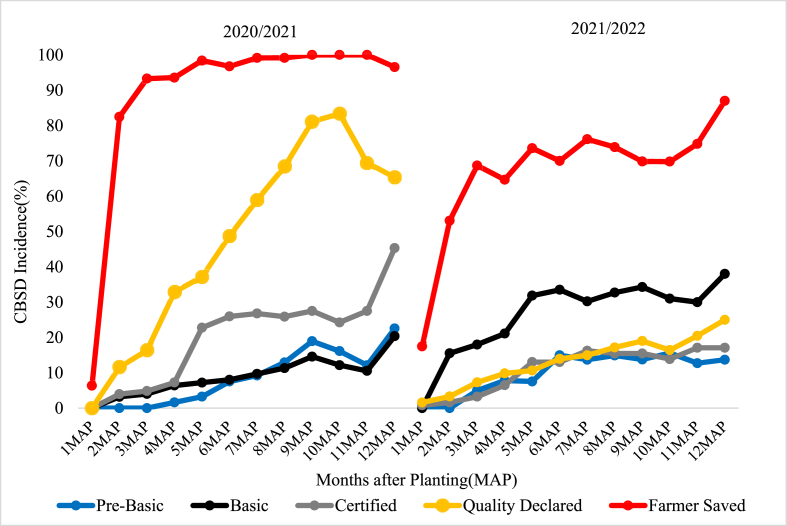
Foliar CBSD incidence recorded at monthly intervals in plots of Mkuranga1 variety for two seasons at Mkuranga site, central-eastern Tanzania.

**Fig. 3:**
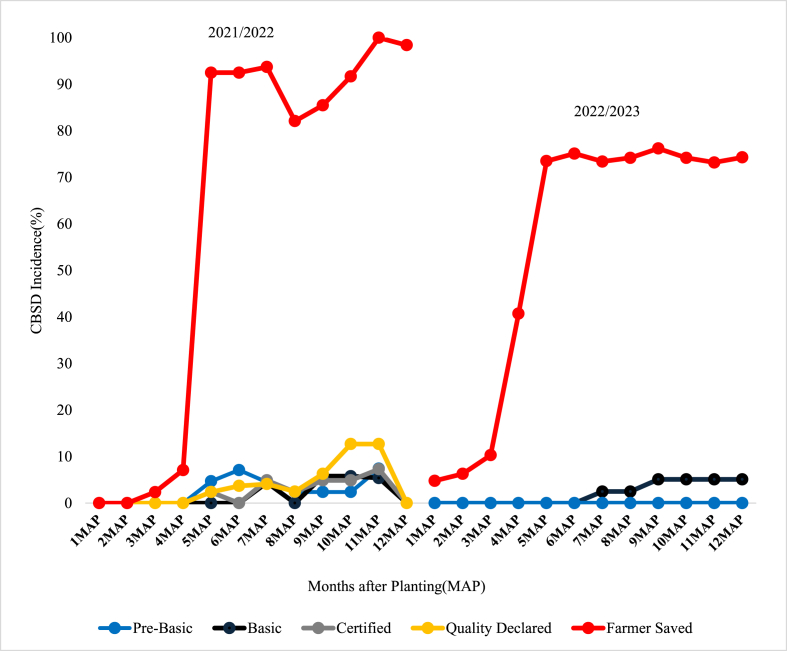
Foliar CBSD incidence recorded at monthly intervals in plots of Mkombozi variety for two seasons at Maruku, north-western Tanzania.

The incidences of CBSD for HQS categories at 1MAP across the varieties and planting seasons were relatively low compared to FSS although these differences were not statistically significant (Fig. 1, Fig. 2, Fig. 3). By 2MAP, however, CBSD incidence for FSS was significantly greater than that in all other seed treatments (p < 0.001) for *Mkuranga1* and *Kiroba*, although there was no significant difference for *Mkombozi* (p > 0.05). By 3MAP, CBSD incidence for *Mkuranga1* and *Kiroba* had peaked at around 90% for FSS, and this changed little until the end of the experiment at 12MAP. The peak CBSD incidence for *Mkombozi* was reached later, from 4-5MAP. For all sites and seasons, the differences in foliar CBSD incidences across varieties between FSS and HQS treatments were greatest from 2-5MAP.

**Fig. 4:**
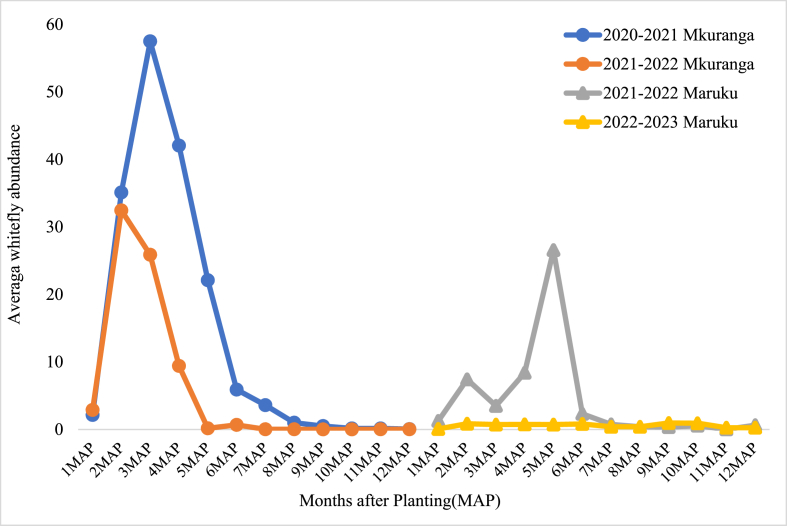
Mean whitefly abundance recorded at monthly intervals at the Mkuranga and Maruku experimental sites over the course of the two-season experiments at each site. Whitefly abundance values were averages each month for all cassava varieties and treatments at each of the sites.

There was an important contrast in the pattern of foliar CBSD disease spread across varieties within HQS plots between Mkuranga and Maruku. Initially, high levels of CBSD were observed in the FSS plots compared to largely healthy plots of HQS at Mkuranga. However, with time, there were steady increases in CBSD foliar incidence at Mkuranga in all treatments over the course of the experiment, while at Maruku, HQS maintained low levels of CBSD incidence until the end of the trial. An important contrast between the sites that was linked to patterns of disease epidemiology was the difference in whitefly abundance (Fig. 4). There were consistently fewer whiteflies at Maruku than there were at Mkuranga. Associated with this, there were significantly higher populations of whiteflies on the varieties at Mkuranga (*Kiroba* = 40.1, *Mkuranga1* = 28.5) than there were on *Mkombozi* (2.9) at Maruku (p < 0.001).

### 3.3 Marketable and non-marketable root number

Marketable root number was lowest for FSS (48.3) and highest for QDS (82.8) (Table 2). There was a statistically significant difference in mean number of marketable roots between all seed classes (p < 0.006) and the mean separation analysis confirmed that marketable root number was lower for FSS than for all of the HQS classes. When considering individual varieties, differences were less clear-cut, but FSS produced fewer marketable roots for *Mkombozi*, and CS for *Mkuranga1* produced significantly more marketable roots than all other seed classes. There were no statistically significant differences in marketable root number between any of the seed classes for *Kiroba*.

**Table 2:** Comparison of marketable fresh root number by seed class and variety using ANOVA.

Varieties	Source of Variation	DF	Sum of Squares	Mean Square	F-value	P-value
**Overall**	Seed class	4	15801	3950	3.85	0.0064**
	Residual	85	87320	1027		
						
	Means separation by least significant difference (LSD)					
	Seed class	Marketable root number	Groups			
	PBS	82.6	a			
	BS	81.7	a			
	CS	76.7	a			
	QDS	82.8	a			
	FSS	48.3	b			
						
**Kiroba**	Seed class	4	3191	797.7	1.01	0.42
	Residual	25	19692	787.7		
						
	Means separation by least significant difference (LSD)					
	Seed class	Marketable root number	Groups			
	PBS	65.5	a			
	BS	62.3	a			
	CS	54.8	a			
	QDS	81.2	a			
	FSS	51.7	a			
						
**Mkuranga1**	Seed class	4	6801	1700.2	2.104	0.11
	Residual	25	20202	808.1		
						
	Means separation by least significant difference (LSD)					
	Seed class	Marketable root number	Groups			
	PBS	86.7	ab			
	BS	69.8	ab			
	CS	92.7	a			
	QDS	57.3	b			
	FSS	55.5	b			
						
**Mkombozi**	Seed class	4	22327	5582	6.49	0.0010**
	Residual	25	21516	861		
						
	Means separation by least significant difference (LSD)					
	Seed class	Marketable root number	Groups			
	PBS	82.6	a			
	BS	81.8	a			
	CS	76.7	a			
	QDS	82.8	a			
	FSS	48.3	b			

### 3.4 Fresh root yield

Overall fresh root yield (FRY) varied significantly between the seed classes (p < 0.001) and was lower for FSS (9.3 t/ha) than it was for the HQS classes (16.8–20.7 t/ha) (Table 3). As for marketable root number, patterns of variation were less distinct for each of the varieties when considered separately. For *Kiroba*, PBS and BS gave greater FRY than the other classes, while for *Mkuranga1* only PBS had a significantly higher FRY. For *Mkombozi*, all of the HQS classes had higher FRY values than the FSS treatment. A comparison of FRY for certified seed (CS) (seed available for sale directly to farmers) with that of FSS demonstrated the large yield increments accrued from planting CS, as well as a consistent pattern in this respect for all of the varieties tested (Table 4). These increments ranged from 6.2 to 10.0 t/ha, representing equivalent percentage increases from 79.5 to 81.3%.

**Table 3:** Comparison of fresh root yield (t/ha) by seed class across varieties using ANOVA.

Varieties	Source of Variation	DF	Sum of Squares	Mean Square	F-value	P-value
**Overall**	Seed class	4	1372	343.0	5.895	0.00031**
	Residual	85	4946	58.2		
						
	Means separation by least significant difference (LSD)					
	Seed class	Fresh root yield	Groups			
	PBS	20.7	a			
	BS	18.4	a			
	CS	16.8	a			
	QDS	18.2	a			
	FSS	9.3	b			
						
**Kiroba**	Seed class	4	280.1	70.0	2.32	0.085
	Residual	25	753.7	30.2		
						
	Means separation by least significant difference (LSD)					
	Seed class	Fresh root yield	Groups			
	PBS	15.7	a			
	BS	16.4	a			
	CS	14.1	ab			
	QDS	13.0	ab			
	FSS	7.8	b			
						
**Mkuranga1**	Seed class	4	296.2	74.1	4.76	0.0054**
	Residual	25	388.9	15.6		
						
	Means separation by least significant difference (LSD)					
	Seed class	Fresh root yield	Groups			
	PBS	17.0	a			
	BS	10.4	b			
	CS	14.0	ab			
	QDS	13.1	ab			
	FSS	7.8	b			
						
**Mkombozi**	Seed class	4	1258.6	314.7	10.1	5.3–05***
	Residual	25	778.8	31.2		
						
	Means separation by least significant difference (LSD)					
	Seed class	Fresh root yield	Groups			
	PBS	29.5	a			
	BS	28.6	ab			
	CS	22.3	b			
	QDS	28.4	ab			
	FSS	12.3	c

**Table 4:** Fresh root yield of certified seed (CS) vs farmer saved seed (FSS).

	Fresh root yield (CS) (t/ha)	Fresh root yield (FSS) (t/ha)	Additional yield from CS (t/ha)	Yield increases attributed to the use of CS (%)
Overall	16.8	9.3	7.5	80.6
Kiroba	14.1	7.8	6.3	80.7
Mkuranga1	14.0	7.8	6.2	79.5
Mkombozi	22.3	12.3	10.0	81.3

### 3.5 CBSD root incidence

There was a significantly higher level of root incidence overall in the FSS treatment (44.8%) compared to the HQS seed classes (4.5–9.9%), although there were significant differences in root incidence for each of the three varieties (Table 5). For variety *Kiroba*, root incidences were low and insignificantly different for each of the seed classes (0.75–2.4%), whilst for both *Mkombozi* and *Mkuranga1*, root incidences of CBSD were much greater in the FSS treatment (65.0% and 67.7% respectively) than they were in the HQS seed classes (3.6–5.8% and 7.6–22.9% respectively).

**Table 5:** Comparison of root CBSD incidence (%) by seed class and variety using ANOVA.

Varieties	Source of Variation	DF	Sum of Squares	Mean Square	F-value	P-value
**Overall**	Seed class	4	20488	5122	13.3	1.9e-08***
	Residual	85	32733	385		
						
	Means separation by least significant difference (LSD)					
	Seed class	Mean-rCBSDInc	Groups			
	PBS	4.54	a			
	BS	8.11	a			
	CS	6.76	a			
	QDS	9.94	a			
	FSS	44.8	b			
						
**Kiroba**	Seed class	4	15.7	3.92	0.61	0.66
	Residual	25	160.3	6.41		
						
	Means separation by least significant difference (LSD)					
	Seed class	Mean-rCBSDInc	Groups			
	PBS	0.75	a			
	BS	2.40	a			
	CS	0.33	a			
	QDS	1.13	a			
	FSS	1.68	a			
						
**Mkuranga1**	Seed class	4	13612	3403	8.44	0.00019***
	Residual	25	10080	403		
						
	Means separation by least significant difference (LSD)					
	Seed class	Mean-rCBSDInc	Groups			
	PBS	7.55	a			
	BS	16.8	a			
	CS	16.4	a			
	QDS	22.9	a			
	FSS	67.7	b			
						
**Mkombozi**	Seed class	4	17311	4328	43.95	5.9e-11***
	Residual	25	2462	98		
						
	Means separation by least significant difference (LSD)					
	Seed class	Mean-rCBSDInc	Groups			
	PBS	5.33	a			
	BS	5.14	a			
	CS	3.59	a			
	QDS	5.84	a			
	FSS	65.0	b			

### 3.6 Usable and unusable fresh root yield (FRY)

There was an overall much higher level of unusable FRY (25.8%) for FSS than for the HQS classes (1.9–3.8%), although this varied greatly between varieties (Table 6). Considering the CS class only, unusable FRY percentages ranged from 0.8% for *Kiroba* to 11.9% for *Mkuranga1*, while for FSS the range was from 1.7% for *Kiroba* to 52.7% for *Mkuranga1*. ANOVA considering all varieties confirmed an overall significant difference in unusable FRY between seed classes (Table 7), with mean separation analysis confirming that unusable FRY for FSS (2.38 t/ha) was significantly greater than that for the HQS classes (0.47-0.72 t/ha). ANOVA also highlighted differences between varieties with respect to unusable FRY as there were no significant differences in unusable FRY between any of the seed classes for *Kiroba*, in contrast to the other two varieties where unusable FRY for FSS was significantly greater than that for all of the HQS classes.

**Table 6:** Comparison of usable and unusable fresh root yield (FRY) (t/ha).

		Pre-Basic	Basic	Certified	QDS	FSS
Overall	Overall FRY	20.7	18.4	16.8	18.2	9.3
	Usable FRY	20.3	17.7	16.2	17.6	6.9
	Unusable FRY	0.4(**1.9**)	0.7(**3.8%)**	0.6(**3.6%**)	0.6(**3.3)**	2.4(**25.8%)**

Kiroba	Overall FRY	15.7	16.4	14.1	13.0	7.77
	Usable FRY	15.7	15.2	14.0	12.9	7.64
	Unusable FRY	0.04	0.36	0.11(**0.8%)**	0.11**(0.8**)	0.13(**1.7%**)

Mkuranga1	Overall FRY	17.0	10.4	14.0	13.1	7.84
	Usable FRY	16.1	8.80	12.4	11.4	3.71
	Unusable FRY	0.94	1.55	1.67(**11.9%)**	1.66(**12.2**)	4.13(**52.7%)**

Mkombozi	Overall FRY	29.5	28.6	22.3	28.4	12.3
	Usable FRY	29.1	28.3	22.3	28.3	9.42
	Unusable FRY	0.42	0.25	0.00(**0%)**	0.16(**0.6**)	2.89(2**3.5%)**

**Table 7:** Comparison of unusable fresh root yield (FRY) by seed class and variety using ANOVA.

Varieties	Source of Variation	DF	Sum of Squares	Mean Square	F-value	P-value
**Overall**	Seed class	4	46.3	11.6	5.74	0.0004***
	Residual	85	171.3	2.02		
						
	Means separation by least significant difference (LSD)					
	Seed class	Unusable FRY(t/ha)	Groups			
	PBS	0.47	a			
	BS	0.72	a			
	CS	0.59	a			
	QDS	0.62	a			
	FSS	2.38	b			
						
**Kiroba**	Seed class	4	0.38	0.096	0.68	0.61
	Residual	25	3.53	0.14		
						
	Means separation by least significant difference (LSD)					
	Seed class	Unusable FRY	Groups			
	PBS	0.04	a			
	BS	0.37	a			
	CS	0.10	a			
	QDS	0.11	a			
	FSS	0.13	a			
						
**Mkuranga1**	Seed class	4	36.5	9.12	3.153	0.032*
	Residual	25	72.3	2.89		
						
	Means separation by least significant difference (LSD)					
	Seed class	Unusable FRY	Groups			
	PBS	0.94	a			
	BS	1.55	a			
	CS	1.67	a			
	QDS	1.66	a			
	FSS	4.13	b			
						
**Mkombozi**	Seed class	4	35.5	8.87	13.66	4.9e-06***
	Residual	25	16.2	0.65		
						
	Means separation by least significant difference (LSD)					
	Seed class	Unusable FRY	Groups			
	PBS	0.42	a			
	BS	0.25	a			
	CS	0.00	a			
	QDS	0.09	a			
	FSS	2.87	b			

**Fig. 5:**
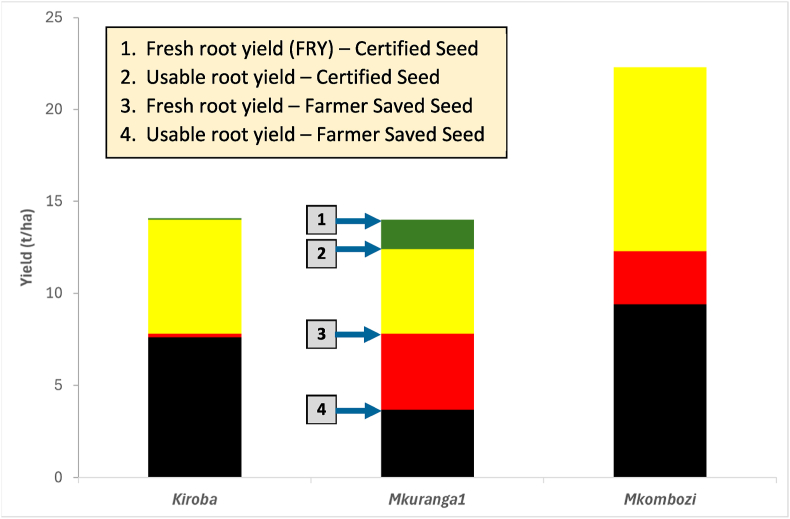
Components of yield loss for cassava varieties in Tanzania, comparing yields derived from certified seed (CS) and farmer-saved seed (FSS). This figure combines data from both seasons at each experimental site.

When considering only usable yield of CS and FSS, overall yield loss was 9.3 t/ha, equivalent to 57.4% (Table 8; Fig. 5). The level of this loss varied between varieties, however, ranging from 12.9 t/ha (57.9%) for *Mkombozi* to 6.4 t/ha (45.7%) for *Kiroba*, although the highest percentage loss was recorded for *Mkuranga1* (70.2%). The pattern of yield loss also varied considerably between varieties (Fig. 5) as differences between total FRY and usable yield differed greatly between *Mkuranga1* (high) and *Kiroba* (low). For *Mkombozi*, there was no difference between FRY and usable yield for CS, which contrasted greatly with FSS where there was a large loss of usable yield due to CBSD root necrosis. Viewing the results overall from the perspective of the yield benefit gained from using CS, the overall yield advantage of planting CS for these three varieties was134.8% compared to planting FSS of the same varieties.

**Table 8:** Comparison between fresh root yield (FRY) and usable FRY for certified seed (CS) and farmer saved seed (FSS) and overall yield loss resulting from the use of FSS (when compared with CS) derived from CS and FSS of three cassava varieties tested at two locations in Tanzania over two seasons. The final column quantifies the overall yield increase to be gained from planting CS, when compared with FSS.

	FRY (CS) (t/ha)	Usable FRY (CS) (t/ha)	FRY (FSS) (t/ha)	Usable FRY (FSS) (t/ha)	Yield loss: CS vs FSS (t/ha)	% Yield loss CS vs FSS	% Yield increase from CS
Overall	16.8	16.2	9.3	6.9	9.3	57.4	134.8
Kiroba	14.1	14.0	7.8	7.6	6.4	45.7	84.2
Mkuranga1	14	12.4	7.8	3.7	8.7	70.2	235.1
Mkombozi	22.3	22.3	12.3	9.4	12.9	57.9	137.2

## 4 Discussion

Certified seed is produced by specialized seed producers under carefully managed conditions, monitored for pests and diseases and its quality is controlled according to the seed laws by seed regulators (Bogdanović et al., 2016). The use of certified seed helps with field disease management which ultimately results in increased crop yield. When yield benefits are quantified, they can be used as an advocacy tool for farmers to adopt the use of certified seed (Bogdanović et al., 2016). There are several studies that have reported the value of certified seed by quantifying the actual yield increment either in percentage terms or as overall yield increases (Clayton et al., 2009), although these have almost entirely focused on cereals and legumes. Very little of this sort of data is available for vegetatively propagated crops. Since there are important on-going efforts to strengthen the sustainability of seed systems for vegetatively propagated crops in several countries across sub-Saharan Africa, the question has arisen – “are there fresh root yield and quality benefits from planting certified seeds compared to farmer-saved seeds of the same variety?” Answering this question was the main basis for the study reported here, where four ‘certified’ seed classes were compared with farmer-saved seed of the same variety. An important locational context for this study in Tanzania is the fact that all cassava-producing areas of the country are affected by cassava brown streak virus disease (CBSD) (Ndyetabula et al., 2016). CBSD affects all currently grown varieties and represents the most important disease constraint for cassava production in Tanzania. The outcomes of this research are likely to be of considerable relevance for other countries in East and Southern Africa which are similarly affected by CBSD.

Seed was sourced for these experiments from FSS which had been recycled for at least five seasons. Since none of the varieties tested have high levels of resistance to CBSD, it was unsurprising that FSS had high levels of CBSD infection at the time of collection. Farmers are typically unwilling to rogue out infected plants in root production fields, and there is clear evidence that CBSD incidence builds up over repeated cropping cycles in areas where CBSD infection is widespread in the environment (Shirima et al., 2019). Seed collected from HQS sources had low levels of CBSD infection, all of which were within the prescribed incidence tolerance levels of the certification system used for HQS of the various categories in Tanzania (Legg et al., 2022).

During the early stages of cassava growth in the experimental trials, the HQS groups had less foliar CBSD incidence compared to FSS (Fig. 1, Fig. 2, Fig. 3). The difference in foliar CBSD incidence between the HQS groups and FSS at that time can be attributed to the difference in initial seed quality between the two seed groups used in the present study. The relatively lower foliar CBSD incidence in HQS groups at the onset of the trial was the result of sourcing seed from certified HQS fields, which immediately demonstrates the value of the management of CBSD which is achieved through virus testing of initial seed stocks, coupled with certification inspections as the seed is propagated down the seed value chain. By contrast, the early season higher incidence of CBSD in plots planted with FSS was clearly linked to the use of FSS cuttings, a high proportion of which were obtained from CBSD-infected stems, recognizing that planting infected cuttings is an important pathway for CBSD spread (Nichols, 1950; Maruthi et al., 2017). Incidences of CBSD have been shown to build up in cassava varieties over serial planting cycles (Shirima et al., 2019), and this study suggested that recycling should not be done for more than 3–4 cycles. In the present study, the FSS had been recycled for between four and six years (Table 1). Cassava farmers in Tanzania recycle their planting material for an average of 7.9 years (J. Yabeja, unpublished data), which is almost double the recommended maximum of four years.

Patterns of increase in CBSD incidence within the experimental plots over the course of the experiments were mainly determined by three factors. The first was the level of infection introduced to the plots through the source material – discussed above; the second was the relative abundance of *Bemisia tabaci* whitefly vectors – which are currently the only known arthropod vector of cassava brown streak viruses (Maruthi et al., 2005, 2017); and the third was the level of infection in neighbouring plots. Where whitefly abundances were high, particularly at Mkuranga, CBSD cutting infection derived from source material acted as a local source of inoculum within experimental plots. Therefore, even though plots planted with HQS initially had low incidences of CBSD, local spread from neighbouring more heavily infected FFS-sourced plots meant that over the duration of the experiment there were large increases in CBSD within plots planted with HQS. Where whitefly abundances were low, as in the second season at Maruku, this effect was greatly reduced, and most plots derived from HQS maintained low levels of CBSD up to the time of harvest. Although cutting-borne infection is an important cause for long distance spread of CBSD, rapid spread of CBSD can occur over short distances where there are high abundances of whitefly vectors (Maruthi et al., 2017). This point highlights the importance of ensuring that new plots planted with HQS are not positioned near to other cassava fields with high CBSD incidences. This point was previously demonstrated through a community-wide phytosanitation programme implemented in north-western Tanzania where initial CBSD-infected stocks of local varieties were replaced with near CBSD-free stocks of an improved variety, leading to sustained reductions in CBSD incidence and greatly increased cassava yields (Legg et al., 2017).

Root necrosis is one of the key symptoms of CBSD (Nichols, 1950), and several studies have demonstrated the link between foliar and root symptoms of CBSD in a quantitative way (Hillocks et al., 1996; Ndyetabula et al., 2016). Although an examination of this link was not part of the current study, CBSD root incidence was greatest in FSS treatments, where foliar incidence was also highest. There was a divergence in this association between varieties, however, as *Kiroba* had a high level of foliar incidence but very little root incidence, in contrast to the two other varieties where foliar and root incidences were closely correlated. *Kiroba* is widely recognized as being a ‘tolerant’ variety in which there is very little root damage even where foliar incidences of CBSD are high (Ndyetabula et al., 2016; Shirima et al., 2019). This is an important reason why it has become one of the most widely-grown varieties in Tanzania and is a key part of the modernized seed system being developed in the country (Legg et al., 2022). Furthermore, this CBSD ‘tolerance’ phenotype expressed by Kiroba is clearly an important trait to be used by breeders in future crossing programmes.

The size of fresh cassava roots determines their market acceptability, with moderate to larger ones fetching higher market value than the small ones, which are more difficult to peel (Nzola et al., 2022).

More important, however, is overall yield, not least since a large proportion of cassava producers continue to produce primarily for home consumption. Our experiments comparing the root production of different sources of cassava seed demonstrated clearly that HQS sources gave significantly greater yields than FSS. A similar pattern was observed for the number of marketable roots produced in the different treatments, demonstrating that most of the increased fresh root yield derived from HQS resulted from the production of a greater number of roots. Differences in both marketable root number and fresh root yield were less clearcut when considering individual varieties, with the exception of *Mkombozi*, most likely due to the lower level of CBSD spread between plots recorded at Maruku where *Mkombozi* was evaluated. The pattern of reduction in fresh root size observed for *Mkombozi* is comparable to other studies that reported diverse losses in cassava roots due to the effects of CBSD, including reduced weight (Hillocks and Thresh, 2000; Nichols, 1950) and number of roots per plant (Ndyetabula et al., 2016).

In the present study, the seed class most widely used by farmers (CS) was used to compute the benefits of cassava FRY arising from HQS when compared to FSS, which is currently the most widely used seed source. Results indicated an overall FRY increase of 80.6%, equivalent to an additional 7.5 t/ha due to the use of CS over FSS of the same improved cassava varieties. With respect to varieties, planting with CS gave yield increases of 80.7% for *Kiroba*, 79.5% for *Mkuranga1*, and 81.3% for *Mkombozi*, when compared with FRY obtained from FSS. An important insight from the current study is that significant yield benefits were obtained in spite of the fact that at the Mkuranga site in particular, planting FSS seed next to HQS almost certainly resulted in relatively higher CBSD incidences in HQS-derived plots than would have occurred if highly CBSD-infected FSS plots had not been planted adjacent to plots planted with HQS. This finding provides evidence that even if farmers plant HQS in the vicinity of neighbouring cassava plants which are infected by CBSD, the use of HQS still guarantees a better yield than would be achieved were they to continue to plant FSS. The increase in FRY due to the use of certified seed revealed in the present study is consistent with other studies that have demonstrated the yield benefits of using high quality seed (Alemu, 2019; Baglan et al., 2020). When considering differences in FRY between the HQS seed groups themselves, there were generally no significant differences. This is not surprising, since the disease tolerance levels for CBSD for each of the seed classes are all relatively low (<10%), and most seed meeting the quality requirements of the different classes will have CBSD infection levels lower that the tolerance limits. In our study, even source seed of the lowest seed class – QDS – had an average CBSD incidence of less than 5%, while the FSS source material had an average CBSD incidence of 78.3%.

Overall, these results suggest that if improved varieties being disseminated through the seed system have their quality controlled – through farmer management as well as with certification inspections from regulatory authorities – CBSD disease incidence can be effectively controlled, the quality of seed can be sustained, and the seed can continue to deliver yields that will be significantly better than those that would be obtained through using FSS. This is an important validation of the quality management processes that are currently being applied for cassava seed in Tanzania.

Severe white or dry brown corky rot (Nichols, 1950; Anon, 2008) renders cassava roots unusable since they become inedible (Gondwe et al., 2003). Results from the present study showed that the level of unusable fresh root yield due to CBSD root necrosis was significantly less for certified seed (0.6 t/ha; 3.6%) than it was for farmer-saved seed (2.4 t/ha; 25.8%). The pattern of these CBSD root necrosis-associated losses, however, varied greatly between varieties, with *Kiroba* being notable in having no significant difference in unusable yield between CS and FSS treatments, in contrast to the two other varieties where there were significantly higher unusable root losses in FSS compared to the CS treatment. This pattern mirrored the variation between varieties and treatments of CBSD root incidence and is a phenotypic indication of important differences in genetic resistance/tolerance characteristics of different cassava varieties (Kaweesi et al., 2014). These results confirm the resilience of *Kiroba* in tolerating CBSD infection over repeated cycles of cultivation and exposure to cassava brown streak viruses, but they also highlight the damaging degeneration that occurs with a variety like *Mkuranga1*, for which carefully managed HQS can produce high yields, but where serial recycling can lead to severe degeneration and large associated yield losses due to root necrosis. It is notable that the degeneration linked losses reported here for FSS of *Mkuranga1* that has been recycled multiple times (4–6), are much greater than those described for the same variety where its seed had only been recycled once (Shirima et al., 2019). *Kiroba* may be more resilient over repeated cropping cycles than the other two varieties, however, *Mkombozi* yielded the most, and quality traits of *Mkombozi* and *Mkuranga1* may be preferred by farmers over *Kiroba* under certain circumstances. It was notable, therefore, that quality management through certification can provide an effective system through which farmers can obtain cassava planting material that will deliver good yields that will be relatively unaffected by CBSD. Further study will be required, however, to determine how these yields decline with each additional year of recycling from the HQS (no recycling) to FSS (4–6 rounds of recycling) treatments tested here.

## 5 Conclusions

Results presented here quantify the gains that farmers can achieve from planting high quality seed that has been carefully managed by seed producers and certified by regulatory authorities. The average fresh yield benefit delivered by certified seed when compared with farmer-saved seed was 81%. However, when CBSD damage to roots rendering them unusable was also considered, the average benefit of certified seed was 135%. This represents a more than doubling of yield and highlights the value that certified seed represents. Using a seed purchase price of US$ 153 for 1ha (Legg et al., 2022), and a farmgate price for fresh roots of Tsh 670,000/t (IPPMedia, 2024) (equivalent to US$ 245/t), the average production gain of 9.3 t/ha from this study would be equivalent to an income gain of US$ 2,279, which is many times the cost of purchasing the certified seed. This clearly represents a substantial potential income gain for cassava producers. Although these results were obtained in Tanzania, they are likely to be of equal relevance in all countries of East, Central and Southern Africa where CBSD is the major production constraint. This emphasizes the need firstly to establish high quality seed systems for cassava, but secondly to raise awareness amongst all stakeholders, and particularly cassava farmers, about the potential of high-quality seed to control disease, increase yield and raise their incomes.

## Funding

Financial support for this work was provided by the 10.13039/100000865Bill and Melinda Gates Foundation, 10.13039/100021038Seattle, WA through the 10.13039/100022898International Institute of Tropical Agriculture (10.13039/100022898IITA) under the *Building Economically Sustainable Seed Systems for Cassava* Phase II (BASICS-II) Project [grant number ***817663***]. Funding support was also received from the Integrated Seed Sector Development (ISSD) Project under the Integrated Seed Sector Development Africa Community of Practice. Contributions of James Legg were supported through the Seed Equal Initiative of the 10.13039/501100015815CGIAR.

## CRediT authorship contribution statement

**Juma W. Yabeja:** Writing – original draft, Methodology, Investigation, Formal analysis, Data curation, Conceptualization. **Mkabwa L.K. Manoko:** Writing – review & editing, Validation, Supervision, Project administration, Conceptualization. **James P. Legg:** Writing – review & editing, Supervision, Project administration, Methodology, Funding acquisition, Conceptualization.

## Declaration of competing interest

The authors declare that they have no known competing financial interests or personal relationships that could have appeared to influence the work reported in this paper.

## Data Availability

Data will be made available on request.
